# Supplementary material for: Personalized Use of an Adjustable Movement-Controlled Video Game in Obstetric Brachial Plexus Palsy during Physiotherapy Sessions at School: A Case Report
Source: Healthcare (Basel). 2023 Jul 12;11(14):2008. doi: 10.3390/healthcare11142008 (PMC10379120; doi:10.3390/healthcare11142008)
Supplement: Supplementary file 1 [file healthcare-11-02008-s001.zip › healthcare-2419447-supplementary.pdf]

Supplementary material

1. Modified Borg scale, perception of the effort:

¿Cómo te sientes de cansado?

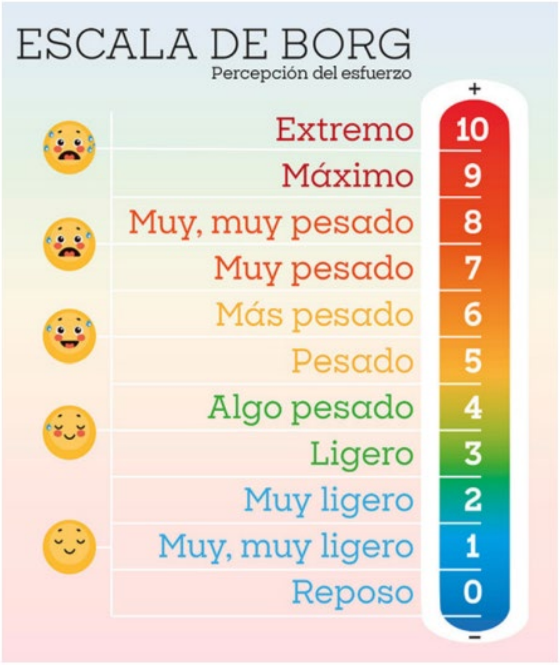

Question: How tired do you feel?

Scales: 10 – extreme, 9 – máximum, 8 – very very heavy, 7 – very heavy, 6 – more heavy, 5 – heavy, 4 – some, 3 – light, 2 – very light, 1 – very very light, 0 – not at all

The figure has been taken from: www.misejercicios.es, Hospital Universitario de Fuenlabrada, 28942 Fuenlabrada, Spain

2. Simple 5 point scale:

¿Cómo te lo has pasado hoy?

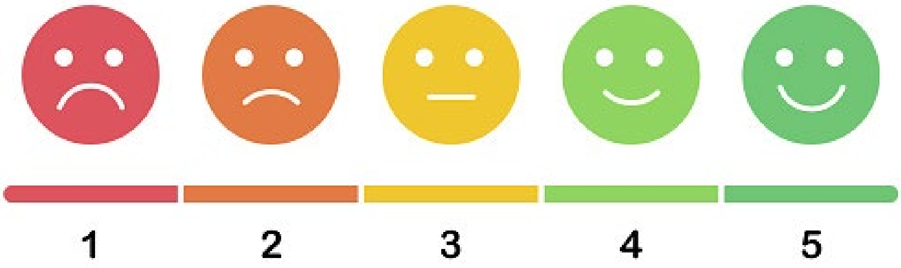

¿Quieres volver a jugar la próxima semana?

Questions: Did you have fun today? Do you want top lay next week again?

3. Visual analogue scale

¿Te duele algo? ¿Cuánto? ¿Dónde?

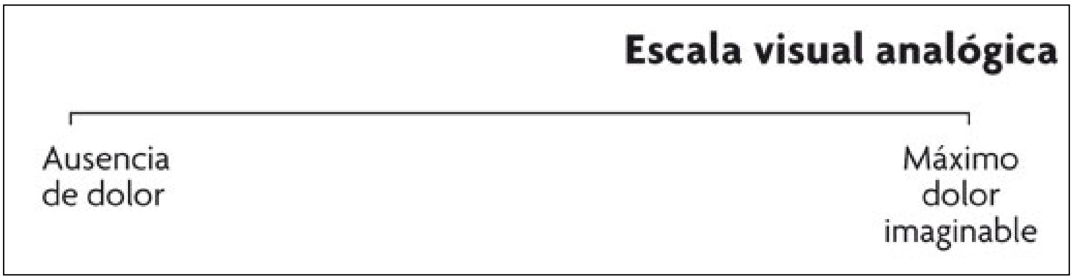

Questions: Do you feel any pain? How much? Where?

Range from left to right: No pain ---- máximum imaginable pain
